# Spatiotemporal variation of endogenous cell-generated stresses within 3D multicellular spheroids

**DOI:** 10.1038/s41598-017-12363-x

**Published:** 2017-09-20

**Authors:** Adam A. Lucio, Alessandro Mongera, Elijah Shelton, Renwei Chen, Adele M. Doyle, Otger Campàs

**Affiliations:** 1 Department of Mechanical Engineering, University of California, Santa Barbara, California USA; 2 California NanoSystems Institute, University of California, Santa Barbara, California USA; 3 Center for Bioengineering, University of California, Santa Barbara, California USA; 4 Neuroscience Research Institute, University of California, Santa Barbara, California USA; 5 Department of Molecular, Cell and Developmental Biology, University of California, Santa Barbara, California USA

## Abstract

Multicellular spheroids serve as an excellent platform to study tissue behavior and tumor growth in a controlled, three-dimensional (3D) environment. While molecular and cellular studies have long used this platform to study cell behavior in 3D, only recently have studies using multicellular spheroids shown an important role for the mechanics of the microenvironment in a wide range of cellular processes, including during tumor progression. Despite the well-established relevance of mechanical cues to cell behavior and the numerous studies on mechanics using 2D cell culture systems, the spatial and temporal variations in endogenous cellular forces within growing multicellular aggregates remain unknown. Using cell-sized oil droplets with controlled physicochemical properties as force transducers in mesenchymal cell aggregates, we show that the magnitude of cell-generated stresses varies only weakly with spatial location within the spherical aggregate, but it increases considerably over time during aggregate compaction and growth. Moreover, our results indicate that the temporal increase in cellular stresses is due to increasing cell pulling forces transmitted via integrin-mediated cell adhesion, consistent with the need for larger intercellular pulling forces to compact cell aggregates.

## Introduction

Cell culture techniques have provided an excellent platform to perform molecular and cell biology studies with carefully controlled biochemical conditions, especially when compared to more complex *in vivo* systems. 2D cell monolayers have been extensively used in cell culture studies, but they rarely mimic tissue-like conditions^1,2^ and, in many cases, display key differences from 3D tissues, such as altered cell morphology, size, gene expression and proliferation^3^. 3D cell culture techniques overcome some of these problems and more closely recapitulate tissue-like physiological conditions, while also allowing high-throughput studies for various applications, including drug testing^4,5^. Multicellular spheroids are 3D aggregates of adherent cells that adopt an overall spherical morphology and display key defining features of 3D tissues through cell-cell and cell-matrix interactions^6,7^. Additionally, chemical gradients established within spheroids (typically larger than 150–200 *μ*m in diameter) due to their 3D geometry make them suitable to model tumor formation, which are often subject to inner hypoxic regions and proliferative gradients^8,9^. In addition to chemical gradients, variations in physical quantities, such as cellular forces, can also play a key role in the formation and homeostasis of 3D multicellular systems^8^.

Both the application of external forces and changes in the mechanical properties of the cellular microenvironment significantly alter cell behavior in 2D cell culture systems^10–15^, as well as during development^16–18^ and disease progression in 3D tissues^19–23^. Most of our understanding of how cells generate forces and respond to mechanical cues was enabled by crucial advances in measuring cell forces in 2D multicellular systems^11–15^ (mainly cell monolayers in culture conditions) and 3D systems with cells embedded in artificial gel matrices^24,25^. In contrast, our understanding of cellular force generation within 3D multicellular systems remains poorly understood. While several studies have focused on the role of the mechanical environment external to multicellular 3D aggregates, the endogenous distributions of cell-generated stresses that build and sustain multicellular aggregate formation and growth in 3D remain unknown.

Internal, endogenous spatial variations in cellular stresses have been suggested to exist within 3D cellular spheroids and tumors^26,27^, but never observed directly. Previous studies estimated internal stresses by physically cutting excised tumors and using the resulting tissue deformation, along with mathematical modeling, to infer these forces^28^. Using this method, compressive stresses within murine tumors were estimated to range from 0.37–8.01 kPa. Less invasive methods have also been used to measure the maximal value of stresses generated by cancerous cell spheroids. The resistance to spheroid growth through use of elastic microcapsules^29^ or gels of varying stiffness^30,31^, as well as osmotic pressure applied to the surface of spheroids^27^, have been used to the measure bulk, isotropic mechanical stresses that spheroids generate on the surrounding space. More recently, elastic microbeads were used to measure effects of mechanical compression on the propagation of isotropic stresses within tumor spheroids^32^. While these studies investigate mechanical stresses within aggregates due to confinement and external compression, the spatiotemporal variations of endogenous stresses during aggregate growth and compaction have not been yet investigated.

A recently developed technique employs oil droplets to measure cell-generated stresses *in situ*, within 3D multicellular systems^33^. The technique relies on fluorescently labelled, cell-sized, oil droplets that are injected between the cells in a multicellular 3D system and used as forces transducers. While these droplets were shown in proof-of-principle experiments to be able to measure cell-generated stresses within cellular aggregates of mammary epithelial cells and tooth mesenchymal cells^33^, the limited control of the physical and chemical properties of the droplets, as well as the reliance on non-commercial components, hindered the practical use of droplets as force sensors in many situations.

Here we introduce a new system of droplet stress sensors based only on commercially-available components and with fully-controlled physical and chemical properties. Using these new droplets, we quantify the spatiotemporal changes in endogenous, cell-generated stresses within growing multicellular spheroids of mouse tooth mesenchymal cells, a cell type previously used in experiments reporting cellular forces^33^. We use a two-surfactant system that enables independent control over the droplet interfacial tension and cell-droplet specific interactions, and allows stress measurements even in chemical environments with varying ionic strengths or in the presence of small surface-active molecules, such as cell culture media. By injecting these new droplets in growing spheroids of tooth mesenchymal cells at different spatial locations and stages, we measure the spatiotemporal changes in endogenous cell-generated stresses. While the magnitude of cell-generated anisotropic stresses does not vary strongly with location inside the aggregate, it considerably increases over time during spheroid growth and compaction. Comparing the stresses measured using droplets coated with and without ligands targeting integrin receptors, we show that pulling forces transmitted via integrin-mediated adhesion increase over time during spheroid compaction.

## Results

### Control of Droplet Interfacial Tension and Cell-Droplet Interactions

In order to use oil droplets as force transducers it is important to control the droplet interfacial tension and prevent uncontrolled interfacial tension variations arising from changes in ionic strength or the presence of a variety of small proteins in the surrounding medium, as the value of the measured cellular stresses relies on its knowledge^33^. In addition, control over the droplet’s interfacial tension enables stress measurements in different systems, such as in multicellular aggregates of different cell types or in distinct regions of embryonic tissues, in which cellular stresses may vary by orders of magnitude.

The originally developed droplet force transducers^33^ controlled the interfacial tension and the cell-droplet interactions using the same surfactant molecule. Using a single surfactant, even in the presence of co-surfactants^33^, presents several problems. Cell-induced surfactant aggregation or extraction of surfactants from the droplet surface can lead to uncontrolled variations in the droplet’s interfacial tension, which may affect the accuracy of mechanical stress measurements. Moreover, the droplet’s interfacial tension needs to be adjusted so that droplet deformations generated by cellular stresses can be readily measured with a good signal. Low droplet interfacial tensions provide better signal-to-noise ratio in the measurement of the droplet shape, as the droplet deformations are higher for lower interfacial tensions. However, very low interfacial tensions can lead to sharp variations in the droplet shape that are difficult to quantify accurately. Since actomyosin-generated tensions largely control cortical mechanics (membrane tension is typically smaller, approximately in the range 0.02–0.2 mN/m^34^) and have been measured to be typically in the range 0.1–1.5 mN/m^35,36^, droplet interfacial tensions in the range 1–10 mN/m enable quantitative measurements with moderate droplet deformations. While this range of interfacial tension can be achieved using custom-made dodecylamine (DDA) co-surfactants, as previously shown^33^, it is preferable to have a readily available commercial surfactant system for this purpose. Ideally, such a new surfactant system would decouple the control of interfacial tension from the control of cell-droplet interactions and also shield the droplet’s interface (especially its interfacial tension) from uncontrolled chemical environments.

**Figure 1:**
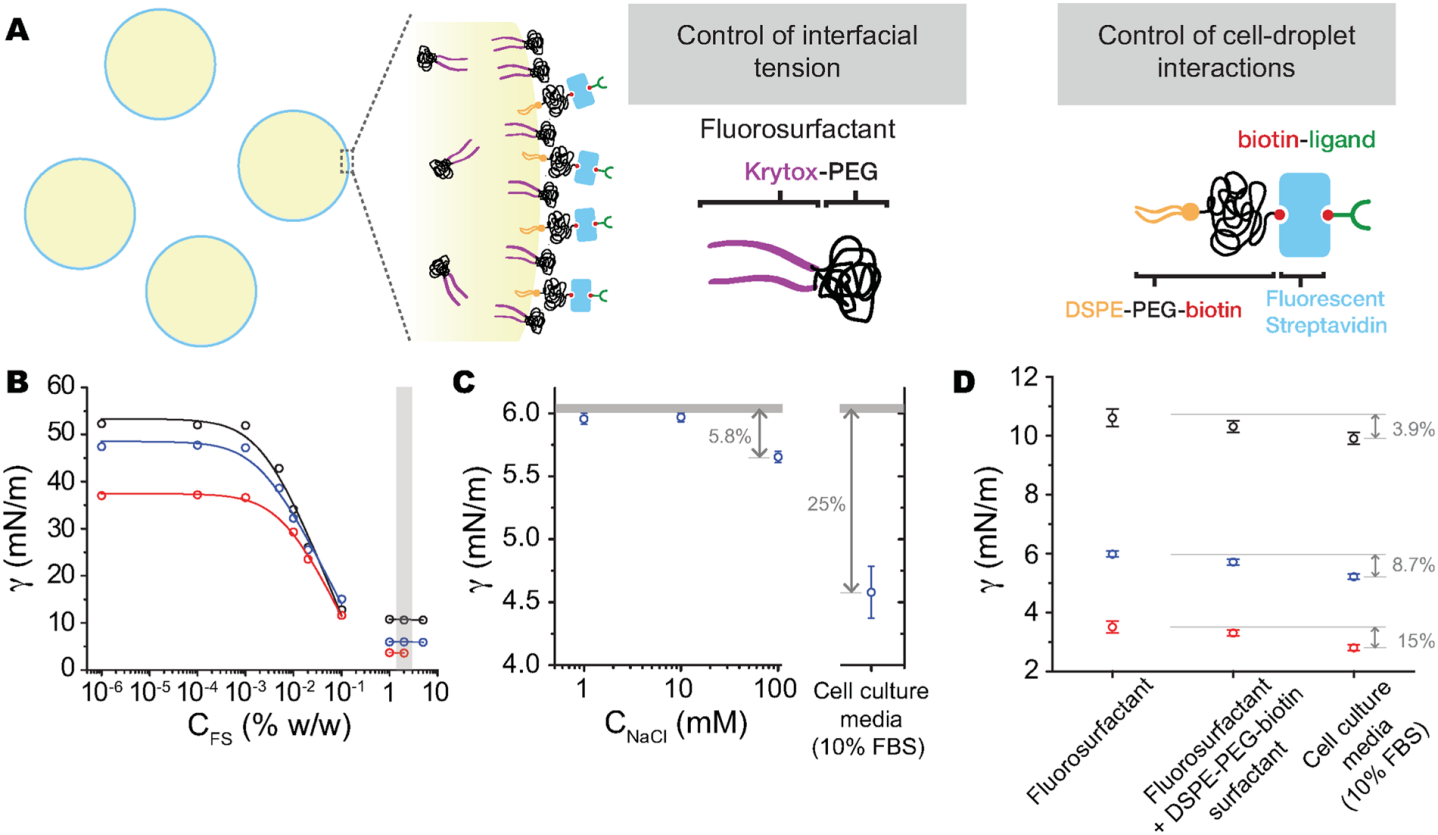
Two-surfactant system to control droplet interfacial tension and cell-droplet interactions. (**A**) Sketch of a droplet and the two-surfactant system used to stabilize and functionalize the droplets. Droplets contain a non-ionic fluorosurfactant (Krytox-PEG) and are coated with a biotinylated surfactant (DSPE-PEG-biotin). The functional biotin group in DSPE-PEG-biotin enables subsequent coatings of the droplets with fluorescent streptavidin conjugates. A biotinylated ligand to control cell-droplet interactions is linked to the streptavidin molecules at the droplet surface. (**B**) Equilibrium interfacial tension of water-fluorocarbon oil (Fluorinert FC43 (black), Novec 7700 (blue) and Novec 7300 (red)) for varying fluorosurfactant concentrations (C_FS_). Fitting the data at low (dilute) fluorosurfactant concentrations (Methods), yields values of interfacial tension in the absence of surfactants (*γ*_*o*_ = 37.9, 48.3, and 53.5 mN/m for Novec 7300, Novec 7700 and Fluorinert FC43, respectively), the saturating surfactant concentration at the interface (Γ_∞_ = 3.51, 4.36, and 4.46 *μ*mol/m^2^ for Novec 7300, Novec 7700 and Fluorinert FC43, respectively), and the Langmuir constant (a_L_ = 6.35, 5.31 and 3.44 *μ*M for Novec 7300, Novec 7700 and Fluorinert FC43, respectively). Linear fits were performed for data in the saturating regime (non-dilute regime), when the interfacial tension saturates to its minimal value. Saturating surface concentrations of fluorosurfactant (2% w/w; gray bar) were used in all other experiments. (**C**) Interfacial tension of Novec 7700 and water, in the presence of 2% (w/w) concentration of fluorosurfactant and varying concentrations (C_NaCl_) of sodium chloride (left) and in cell culture media (right). These values are compared to the value of *γ* in deionized water (gray line). (**D**) Equilibrium *γ* values of fluorocarbon oil (same color code as in B) in water with fluorosurfactant alone (2% w/w) and in the presence of fluorosurfactant in the oil and DSPE-PEG-biotin in the water phase. The interfacial tension of the fluorocarbon oil (Novec 7700), containing fluorosurfactant and coated with DSPE-PEG-biotin, in cell culture media is also shown.

In order to independently control the droplet’s interfacial tension and the droplet-cell interactions, we used a new two-surfactant system composed of a commercially-available fluorinated surfactant, Krytox-PEG(600)^37^ (fluorosurfactant; Fig. 1A), that controls the interfacial tension, and a commercially-available, functionalized phospholipid surfactant, DSPE-PEG(2000)-biotin (Fig. 1A), that mediates the cell-droplet interactions via streptavidin-conjugated ligands targeting cell adhesion receptors at the cell surface. Krytox-PEG(600) is a non-ionic surfactant, composed of fluorinated perfluoropolyether (PFPE) blocks (Krytox) and hydrophilic (PEG) moieties, soluble in the fluorocarbon phase (droplet; Fig. 1A). The droplet interfacial tension can be varied by either changing the concentration of fluorosurfactant (C_FS_) or the type of fluorocarbon oil used (Fig. 1B). Equilibrium interfacial tension measurements were obtained using a pendant drop tensiometer for varying fluorosurfactant concentrations in three different fluorocarbon oils, namely Fluorinert FC43, Novec 7700 and Novec 7300 (Fig. 1B). Increasing the fluorosurfactant concentration decreases the droplet interfacial tension down to a minimal value, which is reached at Krytox-PEG(600) concentrations of about 1–2% (w/w) for the different fluorocarbon oils used. A five-fold decrease in interfacial tension was achieved for FC43, and ten-fold for both Novec 7700 and Novec 7300 (Fig. 1B). The minimal values of the interfacial tension obtained with this surfactant system are as low as 3 mN/m when using Novec 7300 oil, similar to the minimal values of interfacial tension previously obtained using co-surfactants^33^. Importantly, the non-ionic nature of the Krytox-PEG(600) fluorosurfactant allows control of the droplet interfacial tension in environments with strongly varying ionic strengths and helps reduce the adsorption of other small molecules to the droplet interface. Indeed, varying concentrations of ionic strength over several orders of magnitude do not have a substantial effect on the measured interfacial tension (Fig. 1C). For a 2% (w/w) concentration of Krytox-PEG(600) (above the saturation concentration), only very large salt (NaCl) concentrations (C_NaCl_), above approximately 0.1 M, affected the interfacial tension by reducing it slightly (6% reduction for C_NaCl_ = 0.1 M). This small decrease may be either due to the presence of unreacted short PEG molecules or to the presence of a vary small fraction of the ionic unreacted materials used to synthesize Krytox-PEG surfactants^37^. Previous works showed that the interfacial tension of interfaces stabilized only with such ionic materials (charged PFPE surfactants; Krytox-FSH) decreases in the presence of phosphate buffered saline (PBS)^38^. In order to explore how a more complex chemical environment would affect the interfacial tension, we measured it in the presence of cell culture media containing 10% fetal bovine serum (FBS). This chemical environment is characterized by high levels of salts and the presence of several small molecules and proteins, including large concentrations of bovine serum albumin (BSA), a small surface-active molecule. Our measurements indicate that the presence of saturating concentrations (2% w/w) of Krytox-PEG(600) largely shield the interfacial tension, with only a 25% decrease from its measured value in deionized (DI) water. Therefore, the presence of the fluorosurfactant Krytox-PEG(600) alone at saturating concentrations (2% w/w; Fig. 1C) is able to shield the interfacial tension from variations in salt concentrations (ionic strength) and prevent uncontrolled large variations (over 25%) of the interfacial tension in a complex chemical environment such as cell culture media at large FBS concentrations.

In order to control cell-droplet interactions, we further coated the droplets with DSPE-PEG(2000)-biotin surfactants, as previously described^33^. To investigate if the presence of DSPE-PEG(2000)-biotin affects the droplet interfacial tension when the fluorosurfactant is present, we measured the interfacial tension of fluorocarbon droplets containing a 2% (w/w) concentration of Krytox-PEG(600) in the fluorocarbon phase and a high concentration (0.2 mM) of DSPE-PEG(2000)-biotin in the water phase. For all fluorocarbon oils studied, interfacial tension only slightly decreased in the presence of DSPE-PEG(2000)-biotin (Fig. 1D), which could be due to competing adsorption of the two surfactants at the interface. We ruled this out by directly observing the absorbance of DSPE-PEG(2000)-biotin surfactants on the droplet surface in the presence of Krytox-PEG(600) using fluorescence imaging of AlexaFluor-streptavidin conjugates that bind to the biotin groups (see below). We then tested the effect of complex chemical environments on the interfacial tension of droplets coated with both Krytox-PEG(600) and DSPE-PEG(2000)-biotin by incubating them in cell culture media containing a large concentration (10%) of FBS (Methods). In the presence of cell culture media the interfacial tension decreases only slightly, with relative changes in interfacial tension before and after addition of the cell culture media for Fluorinert FC43, Novec 7700 and Novec 7300 of 3.9%, 8.7% and 15%, respectively (Fig. 1D). These results show that while the interfacial tension is barely affected by the addition of DSPE-PEG(2000)-biotin, both surfactants work together to shield the interface from adsorption of small surface-active molecules in the presence of complex chemical environments like cell culture media. Using this two-surfactant system with different fluorocarbon oils (Fluorinert FC43, Novec 7700 and Novec 7300) leads to the same results, albeit with different interfacial tensions. Therefore, different fluorocarbon oils can be used to achieve a desired interfacial tension of the droplet, and the same two-surfactant system can be used in each oil to keep the interfacial tension constant in different chemical environments. While not studied herein, it is possible to vary the density of DSPE-PEG(2000)-biotin on the surface, thereby affecting the surface density of adhesion ligands presented to cells, by changing the DSPE-PEG(2000)-biotin concentration during the formation of droplets. These results demonstrate the versatility of this new, commercial surfactant system, providing low and controlled droplet interfacial tensions even in chemical environments containing high levels of salt and small molecules.

### Control of Droplet Size

**Figure 2:**
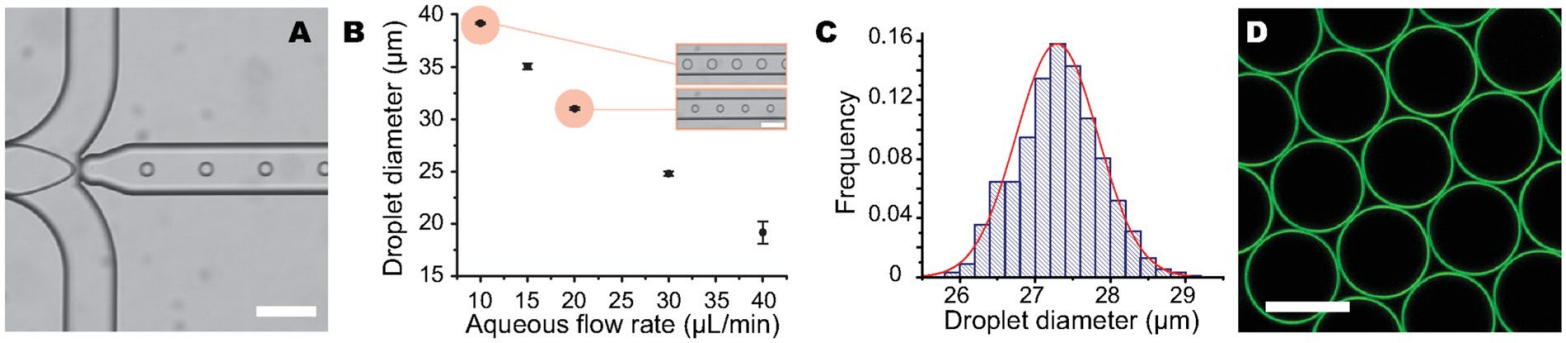
Control of droplet size. (**A**) Droplets generated in glass microfluidic channels, using a flow-focusing device with Novec 7300 oil containing 2% w/w fluorosurfactant as the disperse phase and deionized water with 0.2 mM DSPE-PEG(2000)-biotin as the continuous phase. Scale bar, 100 *μ*m. (**B**) Droplet size range achieved by varying the continuous flow rate while holding the disperse flow rate fixed at 1 *μ*L/min. Inset shows instance of droplet obtained at different flow rates. Scale bar, 100 *μ*m. (**C**) Droplet size distribution of a Novec 7300 (2% w/w fluorosurfactant) emulsion generated with the microfluidic device. Average droplet diameter: 27.3 ± 0.5 *μ*m (mean ± standard deviation). (**D**) Confocal image of a monodisperse emulsion of stable Novec 7300 droplets coated with both fluorosurfactant and DSPE-PEG-biotin, as well as with Alexa-Fluor 633 streptavidin. Scale bar, 20 *μ*m.

The droplet size is an important parameter when measuring cell-generated stresses in multicellular systems. Very small droplets are difficult to deform (due to capillary stresses increasing with decreasing droplet size), whereas droplets much larger than cell size can perturb normal developmental processes and cell-cell interactions. Previous studies have shown that optimal droplet diameters to measure cell-generated stresses are approximately twice the cell size^33^. In many multicellular systems, cell size ranges between 10 and 20 *μ*m, so it is important to be able to generate droplets with controlled diameters in the range of 20–40 *μ*m. To this end, we used droplet microfluidics (Fig. 2A) to generate monodisperse fluorocarbon oil-in-water emulsions of controlled drop size. Droplet microfluidic devices are often fabricated using soft-lithography, taking advantage of the rapid production and low cost of microfluidic devices made from polydimethylsiloxane (PDMS). However, due to their natural hydrophobicity, PDMS-based devices favour water-in-oil emulsions and, without further modification of their surface chemistry, are not optimal to generate fluorocarbon oil-in-water emulsions. Therefore, we employed naturally hydrophilic glass microfluidic devices (Methods) to generate a monodisperse, stable emulsion of fluorocarbon oil-in-water droplets containing both Krytox-PEG(600) and DSPE-PEG(2000)-biotin at the droplet surface (Fig. 2A). This was achieved by using water with DSPE-PEG(2000)-biotin molecules dissolved at a concentration of 0.2 mM as the continuous phase, and Novec 7700 fluorocarbon oil containing 2% (w/w) Krytox-PEG(600) as the dispersed phase. The continuous phase flow rate was set in the range of 30–35 *μ*L/min while the dispersed phase flow rate was fixed at 1 *μ*L/min to produce droplets with a mean diameter of ~20–21 *μ*m. A larger range of droplet sizes can be obtained by further varying the flow rate ratio between the continuous and dispersed phases (Fig. 2B), as previously reported^39^. Droplet sizes generated with this method can be controlled within a 5% relative error (Fig. 2C).

After generation and stabilization of the droplets using microfluidic devices, emulsion droplets were further coated with fluorescent streptavidin molecules using previously established protocols^40^. Confocal images of droplets generated using microfluidics (as described above) and coated with AlexaFluor-streptavidin conjugates show that droplets are uniformly labeled by fluorescent streptavidin molecules, indicating the presence of DSPE-PEG(2000)-biotin surfactants at the droplet interface (Fig. 2D).

### Spatiotemporal Variations in Cell-generated Mechanical Stresses within Cellular Aggregates

**Figure 3:**
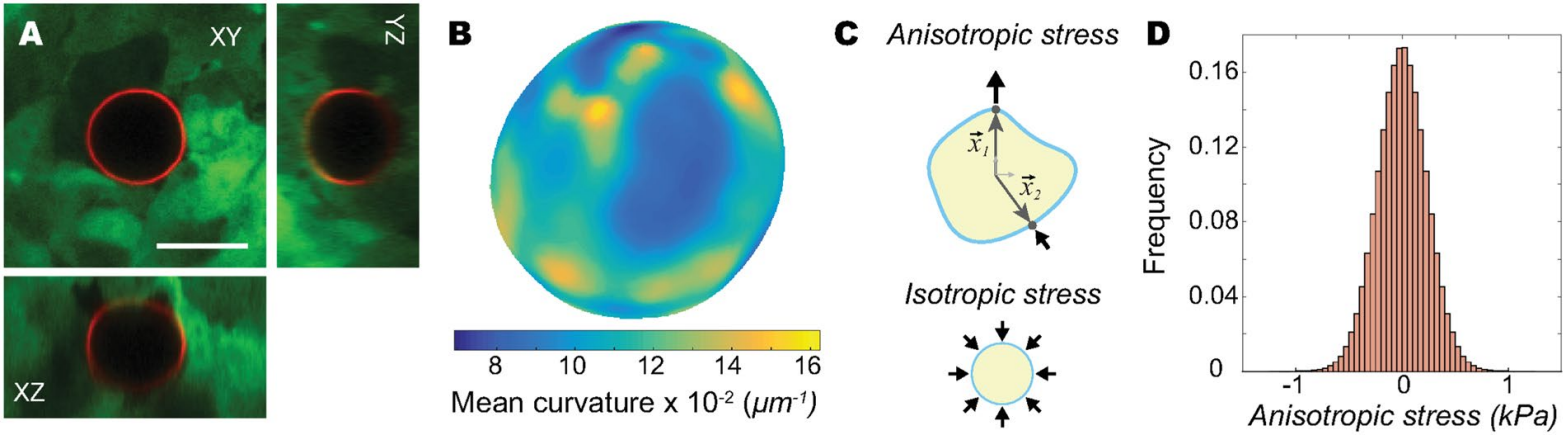
Droplet imaging and 3D reconstruction to measure anisotropic stresses. (**A**) 3D rendering of a confocal z-stack of a droplet coated with Alexa-Fluor 633 streptavidin (red) and RGD peptide embedded within a 3D cell aggregate of tooth mesenchymal cells (green). Scale bar, 20 *μ*m. (**B**) Mean curvature map of the droplet’s surface obtained from 3D reconstruction of confocal images. (**C**) Sketch showing the definition of anisotropic stresses as the difference in normal stresses between two points at the droplet’s surface, each associated with a different spatial direction. While not measured with droplets, a sketch depicting isotropic stresses is also shown for clarity. (**D**) Distribution of measured values of anisotropic stresses for a single droplet and obtained from the stress anisotropy values of every pair of points on the droplet’s surface.

Multicellular aggregates (spheroids) of tooth mesenchymal cells were formed in culture using standard protocols, in non-adherent, round-bottom wells (Methods). In order to measure cell-generated stresses within the aggregate we used the oil droplets described above. By microinjecting droplets at different locations within the aggregate and at different stages (Methods), it is possible to obtain a stereotypical map of spatiotemporal variations in cell-generated stresses as the aggregate grows and compacts. Unless otherwise stated, streptavidin-coated droplets were functionalized with (biotinylated) RGD ligands to target integrin adhesion receptors (as detailed in^40^), enabling the measurement of pulling forces transmitted via integrin molecules in addition to compressive stresses (Fig. 1A). In all cases, the stresses on the surface of each droplet were obtained as previously described^33^ (Methods): first, the droplets within the aggregates were imaged in 3D using confocal microscopy (Fig. 3A) and their surface was reconstructed in 3D. Second, the local mean curvature, H, was calculated at every point of the droplet’s surface (Fig. 3B). Third, we quantified local normal anisotropic stresses, ${\sigma }_{nn}^{A}$, by measuring the differences in normal stresses ${\sigma }_{nn}$ between two different points ${\vec{x}}_{1}$ and ${\vec{x}}_{2}$ on the droplet’s surface, namely ${\sigma }_{nn}^{A}({\vec{x}}_{1},{\vec{x}}_{2})={\sigma }_{nn}({\vec{x}}_{1})-{\sigma }_{nn}({\vec{x}}_{2})$ (Fig. 3C). Local normal force balance at the droplet’s surface establishes the relation between the normal stresses applied to the surface of the droplet, ${\sigma }_{nn}$, and the resulting droplet deformations, characterized by the mean curvature ${\rm{H}}(\vec{x})$, namely^33^

1 $${\sigma }_{nn}(\vec{x})=-{\rm{P}}+2\gamma \,{\rm{H}}(\vec{x})\,,$$

where P is the isotropic pressure (Fig. 3C; not measurable with oil droplets because of droplet incompressibility^33^) and *γ* the interfacial tension of the droplet. Using Eq. (1), the anisotropic stresses between points ${\vec{x}}_{1}$ and ${\vec{x}}_{2}$ on the droplet’s surface are given by

2 $${\sigma }_{nn}^{A}({\vec{x}}_{1},{\vec{x}}_{2})=2\gamma [{\rm{H}}({\vec{x}}_{1})-{\rm{H}}({\vec{x}}_{2})]$$

and depend solely on the droplet’s interfacial tension and the difference of the mean curvature at the two measured points ${\vec{x}}_{1}$ and ${\vec{x}}_{2}$.

In our experiments, we used Novec 7700 oil droplets (coated as described above), which are characterized by an interfacial tension of 5.2 ± 0.1 mN/m in cell culture media with 10% FBS (Fig. 1D). For a given droplet, we obtained the distribution of anisotropic stresses for any two points on the droplet’s surface (Fig. 3D), and used its standard deviation as a measure of the magnitude of anisotropic stresses at the location where the droplet is located within the aggregate. Importantly, the measure of anisotropic stresses defined here is different than in^33^: here we focus on the measurement of stress anisotropy as defined by the difference in stresses along the two spatial directions defined by the normal directions to the droplet at the points ${\vec{x}}_{1}$ and ${\vec{x}}_{2}$ (Eq. (2); Fig. 3C), whereas the measured stresses in^33^ corresponded to the deviations in normal stresses from the isotropic state.

**Figure 4:**
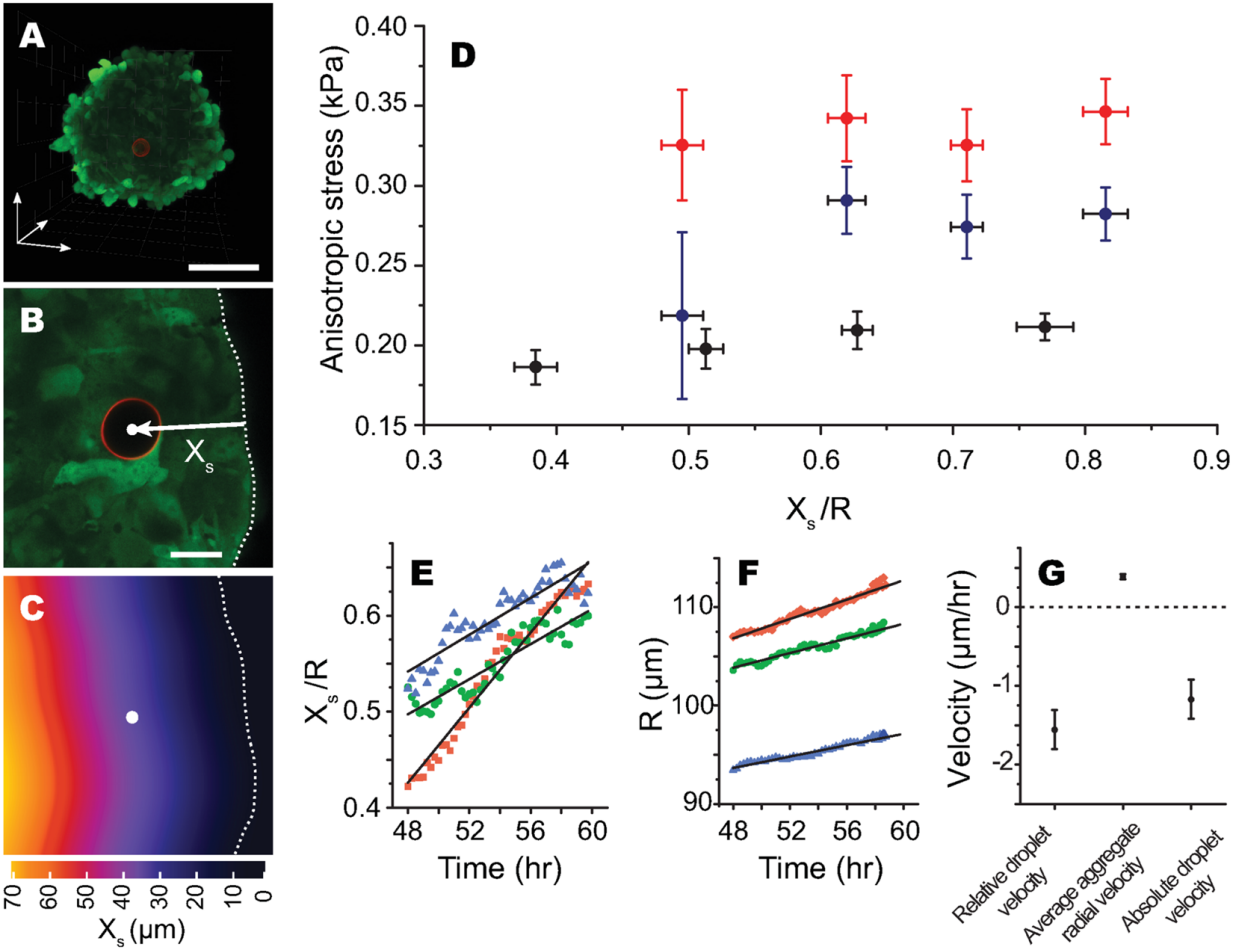
Measurements of spatiotemporal variations in endogenous cellular stresses and droplet movement in 3D tooth mesenchymal cell aggregates. (**A**) Maximum intensity projection of a single droplet (red; surface label) within a 60 hours cell aggregate (green). Scale bar, 100 *μ*m. (**B**,**C**) Definition of X_*s*_ as the closest distance of the droplet center (white circle) from the aggregate’s surface (dashed line), obtained from the contour plot of distance from the aggregate’s surface (**C**). Scale bar, 20 *μ*m. (**D**) Spatial variation in anisotropic stresses within tooth mesenchymal cell aggregates at 24 (black; N = 30), 48 (blue; N = 28) and 60 (red; N = 24) hours (N = number of aggregates). For each time, data points were binned into four groups. Data points and error bars in each bin correspond to mean ± s.e.m of anisotropic stress and *X*_*s*_. For each bin, moving from the leftmost to the rightmost point: N = 4, 8, 13 and 5 (24 hours), N = 4, 7, 10 and 9 (48 hours) and N = 4, 7, 8 and 5 (60 hours). The average aggregate radii for 24, 48, and 60 hours are 98.2 ± 5.7, 103.7 ± 5.8 and 110.2 ± 6.5 *μ*m, respectively (mean ± s.d.). (**E**,**F**) Temporal evolution of droplet position (**E**) and average aggregate radius, *R*, (**F**) for three different aggregates, each containing a single droplet, over 12 hours (from 48–60 hours). In each case, the droplet moves towards the center of the aggregate while the aggregate grows. (**G**) Average droplet velocity relative to the aggregate’s surface (−*dX*_*s*_/*dt*), average aggregate radial growth velocity (*dR*/*dt*) and also the absolute droplet velocity (−*dX*_*s*_/*dt* + *dR*/*dt*). Values reported represent average ± standard error of the mean.

To measure spatial and temporal variations in cell-generated stresses within the aggregate, we injected each aggregate with a single RGD-coated droplet of average diameter 21 ± 1 *μ*m (Fig. 4A), 16 hours after initial cell seeding into round-bottom wells (Methods). We cultured these aggregates for up to 3 days, imaging them and the droplets in their interior at regular intervals of 24, 48 and 60 hours. Given that aggregates are mostly rotationally symmetric, we only report the variations of cell-generated stresses as a function of the minimal distance of the droplet from the aggregate surface (Methods; Fig. 4B,C). Different aggregates contained droplets located at different positions, which allowed us to build a stereotypical spatial map of cell-generated anisotropic stresses by combining the data from all aggregates (Methods; Fig. 4D). The measured magnitude of cell-generated stresses increases only weakly with the distance from the aggregate’s surface at any time point measured. Only about a 10% change in the magnitude of stresses is observed between the measurements closest to the surface and those performed deeper in the aggregate. The spatial range of measureable stresses changes slightly with time because droplets move towards the aggregate core (Fig. 4E), preventing measurements of stresses close to the aggregate’s surface at later times. The existence of inward movement of droplets is consistent with previous observations of core-directed internal flows^41–43^. These flows are thought to be generated by spatial segregation of cell proliferation and death within the aggregate: cell proliferation occurs preferentially at the surface of the aggregate, whereas cell death occurs predominantly at its core, generating a net flow of cells towards the aggregate center^41–43^. We then used the droplets as tracer particles and measured their 3D trajectories over time during aggregate growth from 48 to 60 hours. In all cases, we observed the droplets to move inward, towards the aggregate’s core (Fig. 4E), while the aggregate radius R increased over time (Fig. 4F). Droplets moved inward with an average speed of 1.2 ± 0.3 *μ*m/hr, while aggregates grew at an average radial speed (dR/dt) of 0.38 ± 0.03 *μ*m/hr (Fig. 4G and Methods).

**Figure 5:**
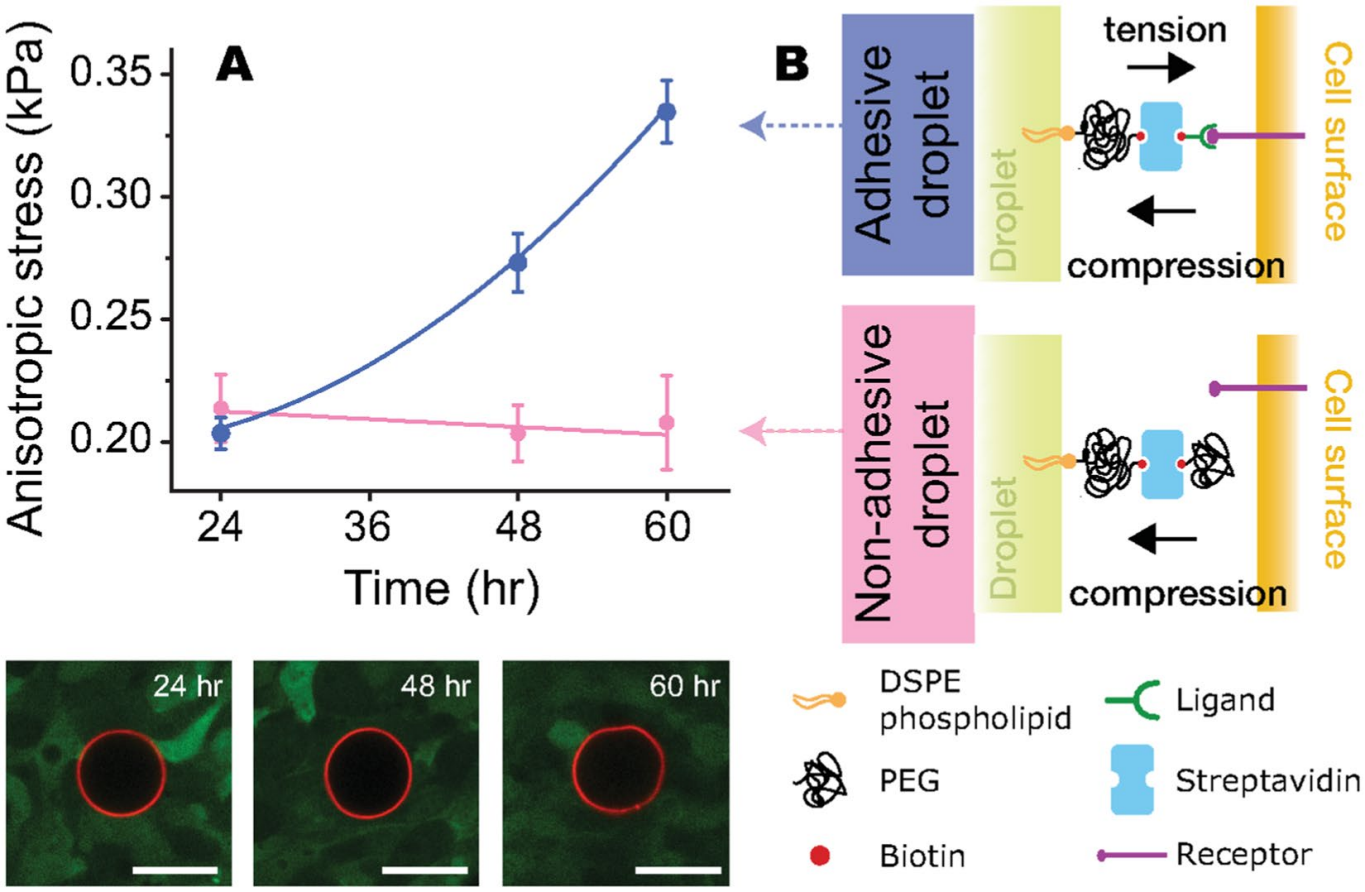
Temporal evolution of average cell-generated compressive and tensile stresses within growing 3D aggregates of tooth mesenchymal cells. (**A**) Average cellular anisotropic stresses for aggregates at 24, 48 and 60 hours (top), measured with droplets coated with RGD peptides (blue circles) or mPEG (orange squares) to quantify compressive and tensile stresses (see panel B). Confocal sections of RGD-coated droplets (red; surface label) embedded between cells (green) of the aggregate at different stages are shown; the increase in the magnitude of droplet deformations (i.e., stresses) over time is directly visible (bottom). Scale bars 20 *μ*m. For 24, 48 and 60 hours, N = 30, 30, and 24, respectively, for droplets coated with RGD peptides, and N = 6, 5, and 5, respectively, for droplets coated with mPEG (N = number of aggregates). (**B**) Schematic representation of adhesive and non-adhesive droplets. Adhesive droplets, coated with ligands targeting cell adhesion receptors (RGD peptide in this case), enable the transmission of pulling cellular forces (tensile stresses), in addition to compressive stresses. Non-adhesive droplets are coated with PEG, preventing cell-droplet adhesion, and only allowing the measurement of (non-specific) compressive stresses.

Despite the weak spatial dependence of cell-generated stresses, their magnitude considerably increased over time (Fig. 4D). Since for a given time point the observed spatial variations in stresses were weak, we averaged them spatially and measured the temporal change in cell-generated stresses over the entire aggregate (Fig. 5A). The magnitude of anisotropic cellular stresses nearly doubles (approximately 0.2 kPa to 0.35 kPa) from 24 to 60 hours of aggregate growth. This increase could be due purely to a temporal increase in (anisotropic) compressive stresses within the aggregate or due an increase in the pulling forces exerted by cells as the aggregate grows and compacts. To differentiate these two scenarios, we also measured the stresses within the aggregate using non-adhesive droplets, coated with methoxypoly(ethylene glycol) (mPEG), that hinder adhesive interactions between the droplet and neighboring cells (Fig. 5A,B). Given that compressive forces on the droplet do not depend on the ability of cells to adhere to it, these mPEG-coated droplets measure only the anisotropic compressive stresses. In contrast, the RGD-coated droplets used above provide a measurement of both compressive and tensile (pulling) stresses. Our results indicate that the magnitude of anisotropic compressive stresses remains constant during aggregate growth (Fig. 5A). Comparing the measurements of RGD-coated droplets and mPEG-coated droplets shows that the measured temporal increase in cell-generated stresses is due to tensile stresses (pulling forces) transmitted through integrin receptors.

## Methods

### Droplet microfluidics to generate fluorocarbon oil-in-water emulsions

Generation of monodisperse fluorocarbon oil-in-water emulsions was achieved using glass microfluidic devices. Borosilicate, flow-focused droplet generators with a 20 *μ*m channel depth were used (Micronit Microfluidics). Single syringe pumps (New Era Pump Systems) were used to deliver fluids at controlled flow rates, through the Fluidic Connect PRO Chip Holder (Micronit Microfluidics), which is custom-made to interface with the flow-focused droplet generator chip. To reduce wetting of droplets to the channel walls, the channels were coated with a 1% (w/w) solution of BSA by flowing it through the channels for 30 min and then subsequently rinsing with deionized water, purified through a Milli-Q purification system (Millipore) for at least 30 min before starting the process of droplet generation. To generate the fluorocarbon oil-in-water droplets, the continuous phase consisted of a solution containing 0.2 mM of DSPE-PEG(2000)-biotin (Avanti Polar Lipids) and the disperse phase consisted of a 2% (w/w) solution of the Krytox-PEG(600) fluorosurfactant (RAN Biotechnologies) in Novec 7700 oil (3 M). Droplets were collected off chip, and subsequently incubated in a glass vial containing a solution of DSPE-PEG(2000)-biotin at a 0.2 mM concentration.

### Fluorescence coating and functionalization of fluorocarbon droplets

Droplets were further made fluorescent and functionalized with a biotinylated ligand for adhesion to cells as described in^40^. Briefly, droplets were placed into a vial containing deionized water and rinsed by replenishing the vial with deionized water a total of three times. Droplets were then aspirated and dispensed via an Eppendorf microloader pipette tip into a swirling (via vortex mixing), aqueous solution of fluorescent-streptavidin conjugates at 2 *μ*M. For all experiments performed with tooth mesenchymal cell aggregates, droplets were coated with streptavidin conjugated with Alexa Fluor 633 (Thermofisher). Droplets were incubated with fluorescent-streptavidin conjugates for at least 1 hour at 4 °C, and then rinsed with deionized water again three times. To mediate adhesion to tooth mesenchymal cells, droplets were coated with a biotinylated arginine-glycine-aspartate (RGD) peptide (Peptides International). To this end, droplets were gently mixed by hand (e.g. rotating the solution in the vial) in a solution of RGD peptides at 20 *μ*M, and subsequently incubated at 4 °C for 1 hour. To hinder adhesion of cells to the droplet surface, droplets were coated with biotinylated mPEG of 1 kDa in molecular weight (Creative PEGworks) by gently mixing them in a solution containing mPEG at 5 *μ*M and incubating them for 1 hour at 4 °C. Finally, droplets were rinsed and stored in a new vial of deionized water at 4 °C.

### Interfacial tension measurements

Measurements of interfacial tension were done using a pendant drop tensiometer, as described in detail in^40^. Briefly, an optical pendant drop tensiometer (Biolin Scientific) was used to obtain equilibrium interfacial tension values. Fluorocarbon oil droplets were suspended from 25 gauge syringe needles (Kimble Chase Life Science and Research Products) and immersed in aqueous media contained in disposable cuvettes (BRAND, 759170). Equilibrium interfacial tension measurements using the fluorosurfactant and phospholipid surfactant were obtained from the tensiometer software (using the Young-Laplace equation) over several minutes to 0.5 hours, depending on the surfactant concentration used. The lab temperature was maintained at 21 °C. Data for equilibrium interfacial tension ${\gamma }_{e}$ versus fluorosurfactant concentration c were fitted (in the dilute regime) using the Gibbs adsorption equation^44^, namely

3 $${\gamma }_{e}={\gamma }_{o}-{\rm{R}}\,{{\rm{T}}{\rm{\Gamma }}}_{{\rm{\infty }}}\,{\rm{l}}{\rm{n}}\,(1+\frac{c}{{a}_{L}})\,,$$

where ${\gamma }_{o}$ is the interfacial tension of the oil-water interface in the absence of surfactants, R is the gas constant, T is the temperature, ${{\rm{\Gamma }}}_{\infty }$ is the saturating surfactant concentration at the interface and a_L_ is the Langmuir constant. For saturating fluorosurfactant concentrations (above the dilute regime), in which the interfacial tension ${\gamma }_{e}$ remains largely constant, we used a linear fit.

### Cell culture and generation of tooth mesenchymal cell aggregates

Mesenchymal cells, isolated from mice tooth rudiments from day 10 post-fertilization embryos (transfected with GFP expressed in the cytoplasm) were a kind gift from Dr. Tada Mammoto (Ingber lab, Wyss Institute for Biologically Inspired Engineering at Harvard). Cells were maintained in Dulbecco’s Modified Eagle Medium (DMEM) supplemented with 10% fetal bovine serum (FBS) and 1% Penicillin Streptomycin (PenStrep), and incubated at 37 °C with 5% CO2. Cell culture media was replenished every other day. Cells were grown to 70–80% confluence before passaging. To generate aggregates, low-attachment, round-bottom well plates were used (Corning). Cells were seeded with 200 *μ*L of culture media at a density of 10^3^ cells/well and placed into incubators. Culture media was replenished every other day by removing 100 *μ*L of old media from round-bottom wells and replacing with 100 *μ*L of fresh media.

### Injection of droplets into aggregates

For injection of droplets into tooth mesenchymal cell aggregates, the aggregates were transferred from the round-bottom well plates to a glass bottom microwell dish (MatTek) with approximately 2 mL of cell culture media. A prepared emulsion with fluorescent, functionalized droplets of Novec 7700 oil was dispensed into the microwell dish next to the aggregates. Injection of droplets at 16 hours after cell aggregation enabled stresses to be measured starting at 24 hours after initial cell seeding. Injection at this early time point does not allow for stress measurements from droplets embedded within aggregates at later time points (typically more than 48 hours, under our experimental conditions), as the fluorescent coating starts to diminish. Injection of droplets at 40 hours enables the measurement of stresses at later time points, namely at 48 and 60 hours in our experiments. In all cases, single droplets were injected using a modification of standard microinjection techniques. Briefly, utilizing a Pico-Liter Injector (PLI-100A, Warner Instruments), a negative pressure was applied to a glass blunt pipette microneedle (20 *μ*m OD; BioMedical Instruments), which was then used to partially intake a droplet at the tip of the microneedle. It should be noted that droplets smaller than 20 *μ*m in diameter would be sucked up into the needle, rather than being held at the tip. In our experiments we found that droplets between 21–23 *μ*m in diameter would avoid this issue. The droplet, held at the tip of the microneedle, was brought inside of the aggregate through a small hole previously made with the same microneedle. Then, the negative pressure was released (and often a slight positive pressure was applied). Upon removing the microneedle, the droplet remained embedded within the aggregate. Aggregates containing droplets were transferred back to the round-bottom well plates and incubated at 37 °C with 5% CO2. To prevent damage from microinjection, we used microneedles with OD of 20 *μ*m, much smaller than the diameter of the aggregates (~200 *μ*m), we performed the microinjections early (when cells are loosely connected) and we let the aggregate recover in culture conditions for at least 8 hours before imaging. No trace of the microinjection event is observed in imaged aggregates with droplets (Fig. 4A).

### Imaging droplets embedded in aggregates

Tooth mesenchymal aggregates containing droplets were transferred from the round-bottom wells using glass pasteur pipette tips (Corning) into a glass-bottom microwell dish (MatTek) containing 2 mL of cell culture media. An inverted Zeiss LSM 710 confocal microscope, equipped with a 40x water immersion lens, was used to obtain 3D fluorescent images of droplets embedded within tooth mesenchymal aggregates. An incubation chamber on the confocal microscope maintained the temperature at 37 °C and 5% CO2 during image acquisition. For long acquisitions of droplet movements over time, aggregates were placed in custom round-bottom wells made from 1% agarose. A PDMS mold was used to create the wells inside the glass-bottom microwell dish. The agarose was heated and transferred to the microwell dish carefully, to avoid the generation of bubbles. The PDMS mold was applied over the agarose and lightly pressed down to create wells close to the glass-bottom surface of the microwell dish. The entire dish was then wrapped in parafilm and stored at 4 °C for at least 30 min. 2 mL of pre-warmed cell culture media was placed into microwell dish before transferring the aggregates into the custom round-bottom wells for imaging.

### Locating droplets within aggregates

In order to obtain the spatial variations of cellular stresses, it is necessary to determine the position of the droplet within the aggregate. The location of the aggregate surface was first obtained from confocal image stacks using Imaris (Bitplane). The same software was used to measure the minimal distance of the droplet center from this surface.

### Image analysis of deformed droplets

Droplet 3D surface reconstruction and analysis of the droplet deformations were done using a similar procedure as that described in^33^. Briefly, the surface of the droplet is first located and described by a point cloud, and the map of mean curvatures on the droplet surface is calculated from the point cloud using standard differential geometry, as described in details in^45^. We used both steerable and median filters (ImageJ plugins) to reduce high-frequency noise in the images before analysis.

## Discussion

We have developed a controlled and versatile system of droplet stress sensors using only commercially-available components. These new droplet sensors enable the measurement of endogenous, anisotropic stresses within multicellular 3D systems, even in chemical environments with varying ionic strengths or in the presence of small surface-active molecules. Using these droplet stress sensors, we measured both the spatial and temporal changes in cell-generated stresses within multicellular aggregates during growth and compaction. These measurements provide a first glance into the spatiotemporal variations in cell-generated stresses within multicellular spheroids, a widely-used model system to study cell behavior in 3D environments.

The new, two-surfactant coatings of the droplets and the use of microfluidics for droplet generation allows full control over the relevant parameters. Despite a slight decrease of interfacial tension in the presence of cell culture media (approximately 8% difference for Novec 7700 fluorocarbon oil), the new two-surfactant system largely shields the interface from variations in ionic strength and the presence of small, surface-active molecules. Previous droplet stress sensors^33^ relied only on ionic surfactants and their interfacial tension was dependent on the ionic strength of the medium surrounding the droplet. The constant and controlled interfacial tension provided by the new two-surfactant system described herein, as well as the ability to functionalize the droplet surface with virtually any biotinylated molecule, enables the measurement of cell-generated stresses in a wide range of systems, including living embryos^18,46,47^, tissue explants^33,46,48^, embryoid bodies^49,50^, organoids^51,52^ and also multicellular spheroids of tumor cells^8,23,32,53^.

The observed weak spatial dependence of the magnitude of anisotropic stresses within the aggregate reveals that the magnitude of cell-generated stresses is similar throughout the aggregate. Comparison of the anisotropic stresses measured with drops coated with and without ligands targeting integrin receptors shows that cells generate increasing pulling, integrin-dependent forces over time, as the aggregate grows and compacts. This may be due to the fact that cells are not able to transmit forces through integrin receptors in loose aggregates (at 24 hours) as easily as in more compact aggregates (above 48 hours). However, several other mechanisms could cause this increase, including a mechanical feedback by which cells could generate larger stresses in response to either increased cellular contacts, increased presence of extracellular matrix or even to changes in the mechanical properties of the cellular microenvironment. Regardless of their origin, these increasing, integrin-mediated pulling forces can substantially contribute to the compaction and cohesiveness of the aggregate. These observations are in agreement with previous experiments showing that integrins mediate strong cohesion between mesenchymal cells in 3D environments^54^. While our measurements compare droplets coated with RGD peptides targeting integrin receptors and non-adhesive droplets (coated with PEG), care must be taken in interpreting the measured temporal increase in anisotropic stresses as being purely caused by integrin-mediated cell-generated forces, as forces from different origins could partially contribute to the droplet deformations and are difficult to rule out.

The magnitude of the average anisotropic stresses is on the order of 0.5 kPa in our experiments. Previous measurements of anisotropic stresses in mesenchymal cell aggregates using oil droplets^33^ reported values of the maximal (rather than average) anisotropic stresses of about 1.5 kPa. Inspection of the distribution of anisotropic stresses measured in our experiments (Fig. 3D) shows that the maximal measured values are approximately 1 kPa, in agreement with previous measurements within experimental error^33^ despite a five-fold difference in the droplet interfacial tensions used in the two works. No other measurements of endogenous, anisotropic stresses have been performed in multicellular spheroids, but several works have estimated the isotropic component of the stresses generated by the entire growing aggregate^29,31^. Tumor spheroids confined in elastic microcapsules have been shown to exert isotropic stresses on the order of 2 kPa^29^, and isotropic stress levels in the range of 3.7–16.0 kPa were estimated within spheroids grown in agarose gels of varying stiffness^31^. While these values are larger than our measurements, it is important to note that these studies measure a different mechanical quantity: the bulk isotropic stress applied by the entire aggregate onto the surrounding medium that confines it. In general, the local stress anisotropy at the cell scale (cell-generated anisotropic stresses) and the global isotropic stresses generated by the entire aggregate are different quantities and should therefore be expected to differ (Fig. 3C). Finally, a recent study used elastic microbeads to measure spatial variations in the transmission of supracellular, isotropic stresses within tumor spheroids under an externally applied osmotic pressure^32^. With an applied surface stress of 5 kPa, the isotropic stresses within the tumor spheroids ranged from <1 kPa to 4 kPa, moving from the periphery to the core. In contrast to the measurements reported herein, which quantify the spatiotemporal variations in the endogenous anisotropic stresses at the cellular scale (Fig. 3C), the measurements described in^32^ correspond to the supracellular, isotropic stresses measured within the aggregate in response to the applied external stress.

The study of mechanical stresses in model *in vitro* systems like multicellular spheroids will help understanding how mechanical cues affect cell behaviour in 3D multicellular environments. Novel tools, such as droplet sensors or elastic microbeads, can provide complementary information about different physical quantities in 3D multicellular environments, complementing our existing knowledge on how molecular cues affect cell behaviour in these systems. Notably, these tools can provide new insights into the role of mechanics in tumor progression.
